# Anti-nephrin antibodies in minimal change disease: Case report series 

**DOI:** 10.5414/CNP104S12

**Published:** 2025-11-28

**Authors:** Maša Knehtl, Nejc Piko, Tadej Petreski, Tadej Zorman, Tina Stropnik-Galuf, Nika Kojc, Karmen Wechtersbach, Robert Ekart, Sebastjan Bevc

**Affiliations:** 1 Department of Nephrology,; 2 Department of Dialysis, Clinic for Internal Medicine, University Medical Center Maribor, Maribor,; 3 Institute of Pathology, Faculty of Medicine, University of Ljubljana, Ljubljana, and; 4 Faculty of Medicine, University of Maribor, Maribor, Slovenia

**Keywords:** minimal change disease, anti-nephrin autoantibodies, nephrotic syndrome, rituximab

## Abstract

Introduction: Minimal change disease (MCD) is one of the causes of nephrotic syndrome (NS) in adults. Recently, anti-nephrin antibodies have been detected in a certain subset of patients with MCD, supporting the proposed autoimmune etiology and appearing to be markers of disease activity. Despite their diagnostic and prognostic potential, the use of anti-nephrin autoantibodies in routine clinical practice is not yet widespread. Studies have shown that patients with anti-nephrin-associated MCD have a more fulminant NS and a better response to antibody-depleting therapy than those who are anti-nephrin-negative. Materials and methods: We report cases of a 79- and a 42-year-old male patient presenting with new-onset NS and acute kidney injury. Both patients had negative immunologic tests, including antibodies against phospholipase A2-receptor (anti-PLA2R) and thrombospondin-7A (anti-THSD7A). Renal biopsy was performed in both patients. Results: In both cases, light microscopy of kidney samples from thick-needle biopsy showed acute tubular injury attributed to severe proteinuria. Electron microscopy revealed diffuse (90%) effacement of the podocyte foot processes without electron-dense deposits. Immunofluorescence presented discrete intracytoplasmic IgG podocyte deposits with a high probability of MCD due to anti-nephrin autoantibodies. In addition to therapy with angiotensin convertase inhibitors, calcium channel blockers, and furosemide, we started treatment with low-dose oral glucocorticoids and mycophenolic acid (case 1) or oral glucocorticoids alone (case 2) depending on patients’ comorbidities. We administered rituximab, resulting in a fast and complete resolution of proteinuria and improvement of kidney function. Conclusion: Anti-nephrin autoantibodies have been detected in a subgroup of patients with MCD supporting the autoimmune etiology of the disease. Targeted anti-B-cell therapy with rituximab is an additional therapeutic option in patients with relapsing or treatment-resistant disease, or drug-related adverse effects to standard therapy.

## Introduction 

Minimal change disease (MCD) is a podocytopathy in which we usually see no specific changes under the light microscope, there are no specific immunofluorescence findings, but under the electron microscopy the characteristic, extensive effacement of the foot processes of the podocytes can be visualized [1]. Recently, autoantibodies against nephrin, a crucial protein of the complex podocyte slit diaphragm architecture, were detected in the serum of a subset of patients with MCD, supporting the autoimmune etiology of the disease and improving our understanding of the efficacy of immunosuppressive therapy for MCD [1, 2]. In addition, the presence of anti-nephrin autoantibodies has also been described in another podocytopathy, primary focal segmental glomerulosclerosis (FSGS) [3]. This finding supports the idea that MCD and primary FSGS are pathophysiologically related and should be categorized under the umbrella term “primary podocytopathies” [4]. In a multicenter study, circulating anti-nephrin autoantibodies were frequently found in patients with MCD and appeared to be markers of disease activity. Among adults, anti-nephrin autoantibodies were found in 44% of patients with MCD and 9% with FSGS. Their binding to the slit diaphragm led to podocyte damage, proteinuria, and consequently to nephrotic syndrome [1]. It was shown that seropositive patients with anti-nephrin-associated MCD had a more fulminant nephrotic syndrome and a better response to antibody-depleting therapy compared to patients who were anti-nephrin-negative [1]. On the other hand, sero-negativization in previously positive patients was associated with clinical remission and a decrease in proteinuria [1]. However, the reliability of conventional immune assays for detecting anti-nephrin antibodies remains controversial [1]. In this article, we present two case reports of adult patients with anti-nephrin autoantibody-positive MCD, their treatment strategies, and outcomes. 

## Case 1 

A 79-year-old man was admitted to our department with new-onset nephrotic syndrome. He developed edema 14 days before admission and noticed reduced urine output. His medical history revealed that these issues began after consuming 1 kg of honey over several days. Clinical examination revealed pretibial and periocular edema. He was normotensive, with a blood pressure of 133/85 mmHg. Laboratory results showed hypoalbuminemia (serum albumin 17 g/L) and hypercholesterolemia (serum cholesterol 6.7 mmol/L). On admission, the serum creatinine level was 165 µmol/L (eGFR 34 mL/min/1.73m^2^), rising to 361 µmol/L (eGFR 15 mL/min/1.73m^2^) over the following days. 

Urine dipstick analysis showed 3+ proteinuria with no hematuria. Urine electrophoresis revealed selective albuminuria (84.8%), and the daily proteinuria was 15 g/day. No monoclonal fraction was detected in the urine. Immunological tests, including ANA, ANCA, anti-dsDNA, and anti-GBM antibodies, were negative, as were antibodies against the phospholipase A2 receptor (anti-PLA2R) and thrombospondin-7A (anti-THSD7A). Ultrasonography showed kidneys measuring 11 × 5.5 cm bilaterally with normal parenchyma and some parapelvic cysts. Chest X-ray revealed pulmonary congestion and a small bilateral pleural effusion. 

During hospitalization, the patient exhibited disorientation and confusion. Mild cognitive impairment had been noted over the past 2 years. Computed tomography (CT) revealed moderate brain atrophy, raising suspicion for mild dementia with a combined etiology (vascular and Alzheimer’s disease). 

**Figure 1:**
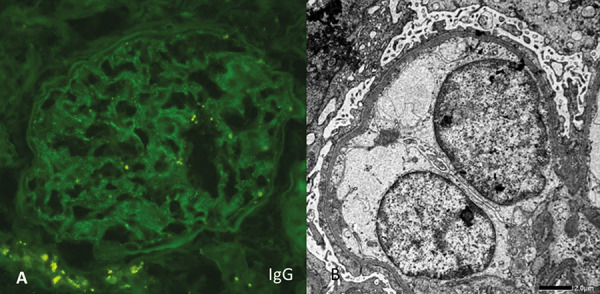
A: Immunofluorescence showing punctate immunoglobulin G along capillary walls. B: Diffuse podocyte processes effacement along glomerular basement membrane.

Kidney biopsy revealed 18 glomeruli, 1 of which showed global sclerosis, while 5% of the tissue showed interstitial fibrosis. Diffuse acute tubular injury, likely secondary to nephrotic syndrome, was noted. Immunofluorescence showed discrete IgG deposits along the glomerular capillary walls and in the podocytes, with a uniform κ/λ distribution, suggesting the presence of anti-nephrin autoantibodies. Electron microscopy revealed diffuse (90%) effacement of the podocyte foot processes without electron-dense deposits (Figure 1). Immunohistochemical staining for NELL1 protein, anti-PLA2R, and anti-THSD7A were negative in the glomeruli. Serum collected after the biopsy and prior to initiating immunosuppressive therapy was positive for anti-nephrin antibodies in qualitative tests, but the quantitative measurement of anti-nephrin antibodies by ELISA was negative. 

An infectious disease specialist was consulted before initiating immunosuppressive therapy due to a positive hepatitis B core antigen test. Serum hepatitis B virus DNA was less than 15 IU/mL, and liver tests and abdominal ultrasound showed no signs of liver disease. Tenofovir was prescribed to address occult hepatitis B infection. Due to a potential pharmacokinetic interaction with tenofovir, the use of calcineurin inhibitors was avoided. The patient initially received furosemide and 20% human albumin infusions, and atorvastatin was introduced. In addition to therapy with the angiotensin-converting enzyme inhibitor perindopril and the calcium channel blocker diltiazem, the patient was treated with low-dose oral glucocorticoids (methylprednisolone 0.12 mg/kg per day) and mycophenolic acid (2 × 750 mg per day). The patient developed steroid-induced diabetes, for which repaglinide was introduced. Bone mineral density was measured using dual-energy X-ray absorptiometry, which revealed osteopenia. In conjunction with methylprednisolone therapy, vitamin D supplementation and calcium carbonate were initiated. 

After 10 days of combined therapy with methylprednisolone and mycophenolic acid, the patient was also treated with rituximab, administered weekly at a dose of 375 mg/m^2^ (700 mg, 700 mg, 600 mg, 600 mg) over 4 weeks. Due to hypogammaglobulinemia, intravenous immunoglobulins (150 mg/kg body weight) were administered before rituximab. 

**Figure 2:**
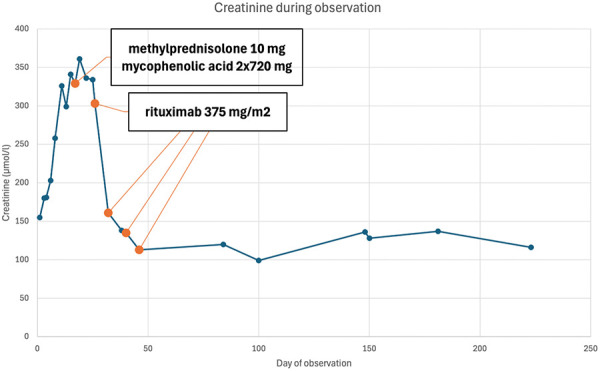
Kidney function of patient 1 over time.

Two months after starting therapy with rituximab, daily proteinuria decreased to below 0.5 g. Serum creatinine fell to 120 µmol/L (eGFR 49 mL/min/1.73m^2^) and normalized in the following month. The patient achieved complete remission. Methylprednisolone therapy was discontinued after 2.5 months. Two months later, the dose of mycophenolic acid was reduced to 2 × 360 mg per day, and after 3.5 months, it was further reduced to 2 × 180 mg per day. No relapse was observed during the 10-month follow-up period. The trend in the patient’s renal function is shown in Figure 2. 

## Case 2 

A 42-year-old man, previously healthy except for a pulmonary embolism following knee surgery a year earlier, which was treated with 6 months of anticoagulant medication, was transferred from a regional hospital to our department due to new-onset nephrotic syndrome. Three weeks before his admission to the regional hospital, he noticed full-body edema and a decrease in urine output, with the urine becoming darker than usual. His body weight increased by at least 10 kg. Over the past month, he had occasionally taken non-steroidal anti-inflammatory drugs (NSAIDs) for intermittent back pain. 

On clinical examination, he had generalized edema. He was 191 cm tall and weighed 130 kg, with a body mass index (BMI) of 35.6 kg/m^2^. In the first few days after admission, he developed high blood pressure, measuring 160/90 mmHg. 

Upon admission, the urine dipstick showed 4+ proteinuria and 20 – 30 erythrocytes per high-power field in the urine sediment. Daily proteinuria was 11.4 g in 500 mL of voided urine. His initial creatinine level was 191 µmol/L, corresponding to an estimated glomerular filtration rate (eGFR) of 36 mL/min/1.73m^2^, as calculated using the CKD-EPI-creatinine equation. Immunological tests (ANA, ANCA, anti-dsDNA, anti-GBM) were negative, including tests for anti-PLA2R and anti-THSD7A. Ultrasound revealed enlarged kidneys, measuring 14 × 6.5 cm bilaterally, with normal thickness and slightly hyperechoic parenchyma. 

On the third day after admission, a kidney biopsy was performed. Kidney biopsy revealed 9 glomeruli, which were all normal under the light microscopy, there was minimal (5%) interstitial fibrosis and collapsed tubular atrophy. Immunofluorescence showed discrete granular IgG deposits along the glomerular capillary walls, suggesting the presence of anti-nephrin antibodies. Electron microscopy revealed diffuse (90%) effacement of the podocyte foot processes. The quantitative measurement of anti-nephrin autoantibodies in the serum, taken prior to initiating immunosuppressive therapy using ELISA, yielded positive results with titers of 291 and 370 RU/mL (cut-off for positivity: 130 RU/mL). 

On the fifth day, after receiving preliminary biopsy results, we started treatment with methylprednisolone at 64 mg daily. Kidney function deteriorated with an increase in creatinine to 361 µmol/L (eGFR 17 mL/min/1.73m^2^) on day 8 after admission. Proteinuria worsened as well, reaching 58 g/day. Once the complete histopathological report confirmed the presence of anti-nephrin autoantibodies, we administered 1 g of rituximab intravenously on day 9 after admission. We also started perindopril 8 mg, empagliflozin 10 mg, and diltiazem 120 mg daily, increasing the diltiazem dose to 180 mg after a few days. 

Laboratory tests showed further worsening of proteinuria, which peaked at 69.8 g/day on day 12. However, proteinuria began to decline thereafter, accompanied by polyuria and the resolution of acute kidney injury. On day 17 after admission, the patient was discharged with a lower body weight of 105 kg (a 25 kg difference), a reduced creatinine level of 108 µmol/L (eGFR 73 mL/min/1.73m^2^), and declining proteinuria (1.80 g per day). His discharge treatment included methylprednisolone 64 mg, dalteparin 10,000 IU/24h subcutaneously, perindopril 8 mg, diltiazem 60 mg 3 times daily, empagliflozin 10 mg, indapamide 2.5 mg, doxazosin extended-release 4 mg, atorvastatin 40 mg, ezetimibe 10 mg, pantoprazole 40 mg, calcium carbonate 1 g, trimethoprim/sulfamethoxazole 80/400 mg, cholecalciferol 8,400 IU/week, and furosemide as needed. 

**Figure 3:**
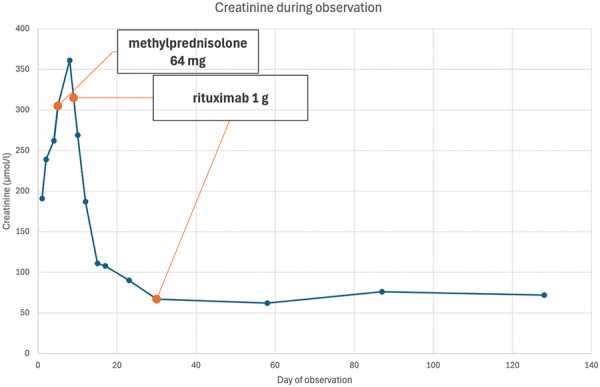
Kidney function of patient 2 over time.

He received the second dose of 1 g rituximab 21 days after the first dose, following a viral respiratory infection, which resolved without specific treatment. After 1 month of treatment, he began tapering methylprednisolone, which was continued for 2 months. Subsequent outpatient follow-up examinations showed that proteinuria had almost disappeared (0.47 g per day) and kidney function had normalized. The trends in the patient’s kidney function are illustrated in Figure 3. 

## Discussion 

Both of our patients presented with new-onset nephrotic syndrome, characterized by severe proteinuria and acute kidney injury, as evidenced by rising serum creatinine levels. Light microscopy during the pathohistological examination of kidney samples from the thick-needle biopsy revealed acute tubular injury, possibly resulting from severe proteinuria, while glomeruli appeared normal. Electron microscopy showed diffuse effacement (90%) of the podocyte foot processes, without electron-dense deposits. Immunofluorescence staining revealed discrete IgG podocyte deposits, strongly suggesting MCD due to anti-nephrin autoantibodies. However, the quantitative assessment of serum anti-nephrin antibodies was only positive in the second patient. Neither patient was considered an ideal candidate for glucocorticoid therapy – the first patient due to advanced age and cognitive impairment, and the second due to a high body mass index. Both patients responded exceptionally well to prescribed combined treatment (the first one with low-dose corticosteroids and MMF + rituximab; the second one with full dose of oral corticosteroids + rituximab), resulting in complete resolution of proteinuria and normalization of kidney function. 

MCD accounts for 10 – 15% of primary nephrotic syndromes in adults [5]. Although spontaneous remission may occur, untreated nephrotic syndrome may lead to increased morbidity, including acute kidney injury, thromboembolic events and infections [5]. Progressive chronic kidney disease is uncommon in patients with steroid-sensitive MCD, while acute kidney injury can occur in association with high-grade proteinuria and hypoalbuminemia [6]. Therefore, the goal of treatment is to achieve and maintain remission. High-dose glucocorticoids are currently the first choice in the treatment of MCD [5]. However, the adverse effects of the drugs remain a major concern for many patients [5]. Prolonged use of glucocorticoids can be harmful, especially in patients with obesity, psychiatric disorders, poorly controlled diabetes, or osteoporosis [6]. 

For patients who cannot tolerate corticosteroid therapy or in whom it is contraindicated, there are other options, including calcineurin inhibitors or mycophenolate mofetil/enteric-coated mycophenolate sodium plus glucocorticoids at reduced doses. Glucocorticoid monotherapy leads to complete remission in 80% to over 95% of adults with MCD [7]. The time course to complete remission varies, with 50% responding within 4 weeks and 10 × 25% requiring treatment for more than 3 × 4 months. However, disease relapse, glucocorticoid resistance, and drug-related adverse events are common problems in adults receiving prolonged high-dose glucocorticoids as first-line therapy for MCD. Numerous observational studies and a small number of randomized trials have shown that ~ 70 or 90% of MCD patients experience complete or partial remission after cyclosporine treatment. Similar results have been reported for tacrolimus. However, over 60% of MCD patients who respond to cyclosporine or tacrolimus relapse after discontinuation of calcineurin inhibitors (usually within 6 months) [7]. 

Rituximab is a monoclonal antibody against the CD20 antigen that binds specifically to B lymphocytes and depletes them. In frequently relapsing or glucocorticoid-dependent MCD, new data have shown that rituximab can reduce relapses and facilitate glucocorticoid withdrawal. Rituximab is currently recommended for recurrent courses of the two primary podocytopathies – MCD and FSGS [6]. However, it is still uncertain whether rituximab alone can or should be used as initial therapy for newly occurring MCD [5]. 

How rituximab exerts its effect in MCD has not been fully explained [5]. The recent discovery of anti-nephrin antibodies as a driver of autoimmunity in a subset of MCD emphasizes the importance of B cells in the pathogenesis of MCD [6]. Fornoni et al. [8] demonstrated that rituximab could contribute to the stabilization of the podocyte cytoskeleton and prevent apoptosis by interacting with the protein sphingomyelin phosphodiesterase acid-like 3b expressed in glomerular epithelial cells. This observation suggests that rituximab therapy may be effective not only by depleting CD20 lymphocytes but also by modulating podocyte function [8]. 

There is evidence that rituximab is effective in the treatment of MCD in adults [7]. In a systematic review and meta-analysis of 21 studies involving 382 adults with frequently recurrent/glucocorticoid-dependent MCD or FSGS, rituximab therapy resulted in complete remission in 92% of patients with MCD, although 28% of patients relapsed during follow-up [9]. Rituximab was well tolerated and was associated with few adverse effects [7, 9]. In the study by Guan et al. [5]the effect of rituximab was investigated in 9 adult patients with new-onset MCD. Sustained remission was achieved in 6 patients, suggesting that rituximab may be a potential alternative for induction therapy in MCD patients, especially for those who have contraindications for glucocorticoid therapy. This is supported by a recent case series in which treatment with 4 weekly doses of rituximab successfully induced complete remission without relapse in all 6 new-onset MCD patients with a follow-up period of up to 36 months [5]. The retrospective study by Zhang et al. [7], which involved 33 patients (22 of 33 patients had relapsing-remitting MCD), suggests that low-dose rituximab significantly reduces the relapse rate and corticosteroid dose in MCD patients. Rituximab, which is associated with prolonged remission, may have remarkable efficacy in the treatment of relapsing MCD in adults and may be favored in patients at high risk of developing adverse effects from corticosteroids [7]. The study by Chebotareva et al. [4] showed that anti-nephrin autoantibodies are associated with the severity of podocytopathies in patients with MCD and primary FSGS. However, they had no influence on the response to immunosuppressive therapy with corticosteroids or cyclosporine. They did not observe remission in FSGS patients with elevated anti-nephrin antibodies with rituximab treatment [4]. A recent study presented by Hengel et al. [1] showed that in 3 patients with anti-nephrin-associated podocytopathy, B-cell depleting therapy with rituximab was able to deplete anti-nephrin autoantibodies and achieve clinical remission. 

Our case series demonstrates that, although histological findings were suggestive of anti-nephrin antibody involvement in both patients, circulating antibodies were detectable in only 1 case. This raises the possibility that currently available ELISA assays may lack sufficient sensitivity for reliably identifying anti-nephrin antibodies. 

The second notable finding is that rituximab was equally effective in the patient with a negative ELISA result, indicating that therapeutic response may not strictly depend on serological confirmation of anti-nephrin antibodies. 

Third, we observed a remarkably rapid resolution of acute kidney injury following rituximab therapy, representing a favorable clinical outcome. This observation is particularly relevant, as such rapid improvement in acute kidney injury is infrequently described in the literature [10, 11]. 

A key limitation of this report is the small sample size, comprising only two cases. Further studies involving larger cohorts are needed to better evaluate the efficacy of rituximab in managing acute kidney injury associated with MCD. 

## Conclusion 

Anti-nephrin autoantibodies have been identified in a subset of patients with MCD and FSGS. These autoantibodies can be detected both in renal biopsies and in the serum of affected individuals. Introducing targeted anti-B-cell therapy may improve the prognosis for these patients. However, the diagnostic and prognostic value of anti-nephrin antibodies in routine clinical practice is not yet fully established, and the standardization of the methodology for detection remains to be determined. 

## Compliance with ethical standards 

Both patients signed an informed consent for the publication of anonymized data. 

## Authors’ contributions 

MK, NP, and TP drafted the manuscript. TP designed Figures 2 and 3, while NK and KW designed Figure 1. MK, NP, and TP conducted the literature review. NP, TZ, TSG, NK, KW, RE, and SB critically revised the manuscript and approved the final version for publication. 

## Funding 

None. 

## Conflict of interest 

The authors declare no conflict of interest.
